# Discriminatory Role of Detergent-Resistant Membranes in the Dimerization and Endocytosis of Prostate-Specific Membrane Antigen

**DOI:** 10.1371/journal.pone.0066193

**Published:** 2013-06-19

**Authors:** Sonja Schmidt, Birthe Gericke, Giulio Fracasso, Dunia Ramarli, Marco Colombatti, Hassan Y. Naim

**Affiliations:** 1 Department of Physiological Chemistry, University of Veterinary Medicine Hannover, Hannover, Germany; 2 Department of Pathology and Diagnostics, University of Verona, Verona, Italy; University of Sassari, Italy

## Abstract

Prostate-specific membrane antigen (PSMA) is a type-II membrane glycoprotein that was initially identified in LNCaP cells. It is expressed at elevated levels in prostate cancer. In view of the correlation between the expression levels of PSMA and disease grade and stage, PSMA is considered to be one of the most promising biomarkers in the diagnosis and treatment of prostate cancer. In LNCaP cells PSMA undergoes internalization via clathrin-coated pits followed by accumulation in the endosomes. PSMA associates with different types of detergent-resistant membranes (DRMs) along the secretory pathway. Its mature form is mainly insoluble in Lubrol WX, but does not associate with Triton X-100-DRMs. To understand the mechanism of PSMA internalization we investigated its association during internalization with DRMs. For this purpose, internalization was induced by antibody cross-linking. We demonstrate at the biochemical and cell biological levels that: [i] exclusively homodimers of PSMA are associated with Lubrol WX-DRMs, [ii] antibody-induced cross-linking of PSMA molecules results in a time-dependent partitioning into another DRMs type, namely Triton X-100-DRMs, and [iii] concomitant with its association with Triton-X-100-DRMs internalization of PSMA occurs along tubulin filaments. In a previous work (Colombatti et al. (2009) PLoS One 4: e4608) we demonstrated that the small GTPases RAS and RAC1 and the MAPKs p38 and ERK1/2 are activated during antibody cross-linking. As downstream effects of this activation we observed a strong induction of NF-kB associated with an increased expression of IL-6 and CCL5 genes and that IL-6 and CCL5 enhanced the proliferative potential of LNCaP cells synergistically. These observations together with findings reported here hypothesize a fundamental role of DRMs during activation of PSMA as platforms for trafficking, endocytosis and signalling. Understanding these mechanisms constitutes an essential prerequisite for utilization of PSMA as a therapeutically suitable target in prostate cancer.

## Introduction

Adenocarcinomas of the prostate are amongst the most common malignancies in men in developed countries. Conventional treatment like prostatectomy or radiation can be curative only if prostate cancer is diagnosed at an early stage.

Prostate-specific membrane antigen (PSMA) is a type-II-transmembrane-glycoprotein with folate hydrolase and carboxypeptidase activity [1], found initially in LNCaP cells by immunoprecipitation [2]. PSMA is expressed in epithelial cells of the prostate and at low levels also in some other organs like kidney, intestine and brain [3], [4]. Elevated levels of PSMA are detected in prostate cancer cells including those that are metastatic [5], [6]. Levels of PSMA are directly proportional to disease grade and stage [7]. Also in neovasculature of other non prostatic tumors PSMA expression has been detected, but it is absent from healthy vasculature [8], [9].

As a consequence PSMA is one of the most promising biomarkers in the diagnosis and treatment of prostate cancer. Antibodies conjugated to cytotoxic drugs are currently in clinical trials for use in mAb mediated immunotherapy [10], [11], [12], [13], [14]. Different specific mAbs conjugated to cytotoxic drugs have shown the ability to induce apoptosis, especially in cells expressing high levels of PSMA on their surface, like prostate cancer cells.

In LNCaP cells PSMA undergoes internalization via clathrin-coated pits followed by accumulation in endosomes [15]. Furthermore PSMA associates with the actin cross linking protein filamin A and this association is involved in the localization of PSMA to the recycling endosomal compartment [16]. In endothelial cells internalization of PSMA is caveolae-dependent and an interaction with caveolin 1 could be detected [17]. Rajasekaran et al. could demonstrate that the cytoplasmic tail five N-terminal amino acids MXXXL are sufficient to mediate the internalization of PSMA [18].

However the function of PSMA, the direct correlation between its expression and increasing tumor aggressiveness in prostate cancer and details about internalization still remain unclear.

To further understand the mechanism of PSMA internalization we investigated the association of PSMA during internalization with lipid rafts or detergent-resistant membranes (DRMs). Lipid rafts are described as dynamic, nanoscale, sterol-sphingolipid-enriched, ordered assemblies of proteins and lipids, in which the metastable raft resting state can be stimulated to coalesce into larger, more stable raft domains by specific lipid-lipid, protein-lipid and protein-protein oligomerizing interactions [19]. These rafts are involved in signalling processes, trafficking and endocytosis.

Extraction with distinct detergents allows isolation of DRMs with different composition [20], [21]. Triton X-100-DRMs are enriched in sphingolipids and cholesterol, whereas Tween 20-DRMs as well as Lubrol WX-DRMs show decreased amounts of these two lipids. In contrast phosphatidylethanolamine is increased approx. 6- and 8-fold in Tween 20- and Lubrol WX-DRMs respectively [21].

Along the secretory pathway PSMA is transported via different microdomains. After interacting with Tween 20-insoluble microdomains and as soon as PSMA enters the Golgi, it associates with Lubrol WX-DRMs and this interaction is either maintained or restored once it reaches the plasma membrane [21]. It is important to note that under normal physiological conditions PSMA does not associate with Triton X-100-DRMs along the secretory pathway [21].

To understand the mechanism of PSMA internalization we investigated its association during internalization with lipid rafts or detergent-resistant membranes (DRMs). For this purpose we induced internalization by antibody cross-linking of PSMA and demonstrate that [i] exclusively homodimers of PSMA are associated with Lubrol WX-DRMs, [ii] antibody-induced cross-linking of these PSMA molecules results in a partitioning of PSMA and α-tubulin into another DRMs type, namely Triton X-100-DRMs and [iii] concomitant to its association with Triton-X-100-DRMs internalization of PSMA occurs along tubulin filaments.

In a previous work we demonstrated that the small GTPases RAS and RAC1 and the MAPKs p38 and ERK1/2 are activated during the process of activation [22]. As downstream effects of the activation we observed a strong induction of NF-kB activation associated with an increased expression of IL-6 and CCL5 genes and that IL-6 and CCL5 enhanced the proliferative potential of LNCaP cells synergistically. In light of these previous observations and our novel findings reported in this manuscript we hypothesize a fundamental role of DRMs during activation and internalization of PSMA as platforms for signalling, trafficking and endocytosis. Understanding these mechanisms constitutes an essential prerequisite for utilization of PSMA as a therapeutically suitable target in prostate cancer.

## Materials and Methods

### Reagents and Antibodies

Tissue culture material was purchased from Sarstedt, RPMI-Medium and Dulbecco’s Modified Eagle’s Medium and supplemented reagents (penicillin-streptomycin, fetal calf serum) were from PAA Laboratories. Triton X-100, proteinase inhibitors, protein A-sepharose and bovine serum albumin were from Sigma. Acrylamide, TEMED, SDS, Tris, sodium chloride, potassium chloride, sodium hydrogen phosphate, potassium di-hydrogen phosphate, sodium deoxycholate, paraformaldehyde, saponin, chaps, glutathione, urea, thiourea, DTT and PVDF membranes were purchased from Carl Roth. The ECL plus Western Blotting Detection System and Biotin were from Thermo Scientific. Lubrol WX was from MP Biomedicals, ammonium chloride from Merck and Coomassie blue G-250 from Serva.

The anti-PSMA mAb D2B recognizing a luminal epitope of PSMA was produced in our laboratory [22]. As previously shown [22] the mAb D2B recognizes human PSMA with the same specificity and somewhat greater affinity than the PSMA specific mAb J591 [15]. 7e11c antibody recognizing a cytosolic epitope of PSMA was purified from an ATCC hybridoma. Horseradish peroxidase-coupled goat anti-mouse antibody was from Dako. The anti-tubulin antibodies were from Sigma and secondary antibodies coupled to Alexa Flour dyes were purchased from Invitrogen. The anti-flotillin-2 and anti-caveolin-1 antibodies were from Santa Cruz Biotechnology. The anti-EEA1 antibody was purchased from Affinity BioReagents.

### Cell Culture

All cell lines used were from ATCC. LNCaP cells and CHO cells were cultured in humidified atmosphere containing 5% CO_2_ in air at 37°C in RPMI-Medium containing 2 g/L glucose in the presence of fetal calf serum (10% v/v) and penicillin-streptomycin (100 U/ml and 0.1 mg/ml respectively). COS-1 cells and MDCK cells were cultured under equal conditions in Dulbecco’s Modified Eagle’s Medium (DMEM) containing 1 g/L glucose, fetal calf serum and penicillin-streptomycin.

### Activation of PSMA by Antibody-induced Cross-linking

Activation of PSMA by antibody-induced cross-linking was performed according to the protocol described in [22]. Briefly LNCaP cells that reach 70% confluence were incubated with 5 µg/ml of the appropriate mAb for 45 minutes at room temperature, washed and placed at 37°C for 15 minutes with 10 µg/ml goat-anti-mouse antibody to induce the clustering of PSMA molecules.

### DRM Extraction

LNCaP cells that reach 70% confluence were washed twice in ice-cold PBS and solubilized in PBS containing 1% (w/v) detergent (Lubrol WX or Triton X-100 respectively) supplemented with a mixture of proteinase inhibitors. Cells were homogenized with a Luer-21 Gauge needle 20times/ml and then maintained on ice for 2 hours. Afterwards, samples were pre-centrifuged 15 minutes at 8.000×g and cell debris was discarded followed by a centrifugation at 100.000×g, 4°C for 90 minutes (Beckman Optima LE-80, SW 55Ti-rotor). The supernatant and pellet obtained, corresponding to the soluble and insoluble fractions respectively, were separately analyzed. The pellet fractions were lysed over night in a lysis-buffer containing 25 mM Tris, 50 mM sodium chloride, 0.5% (w/v) Triton X-100 and 0.5% (w/v) sodium-deoxycholate supplemented with a mixture of proteinase inhibitors. On the next day the protein amounts of all fractions were determined using Bradford protein assay (Bio-Rad) and 50 µg protein of each fraction was analyzed by SDS/PAGE and western blotting.

### Immunoassays

PSMA was immunoprecipitated from supernatant and pellet fractions after DRM extraction from LNCaP cells. Immunoprecipitation was performed by using D2B mAb for 1 hour at 4°C. Antigen-antibody complexes were then recovered with protein A-Sepharose over night, denatured and loaded on SDS/PAGE followed by western blot analysis.

Specific bands on PVDF membranes detected by the appropriate antibodies were visualized by a ChemiDoc XRS Molecular Imager (Bio-Rad) device. Digital images obtained were quantified by using image processing and analysis software ImageJ.

For indirect immunofluorescence, cells were fixed in 4% paraformaldehyde in PBS. Quenching was performed twice for 10 minutes in 50 mM ammonium chloride in PBS. Blocking and addition of the antibodies were performed in PBS containing 1% bovine serum albumin and 0.5% saponin. Preparations were visualized using a Leica TCS SP5 confocal laser microscope with an×63 oil planapochromat lens (Leica Microsystems, Wetzlar).

### Analysis of the Quaternary Structure

Monomers and dimers of PSMA were separated by sucrose density gradients under native conditions. LNCaP cells were lysed for 2 h in PBS containing 1% of Triton X-100 supplemented with a mixture of proteinase inhibitors. After a centrifugation step at 8.000×g to remove cell debris, cell lysates were loaded on top of 5–25% (w/v) continuous sucrose gradients which were centrifuged at 100.000×g for 18 h at 4°C (Beckman Optima LE-80, SW 40Ti-rotor). 24 fractions of 500 µl each were collected from bottom to top. Half of each fraction was precipitated using ethanol and PSMA was detected by western blot analysis.

For analysis of the quaternary structure of PSMA in Lubrol WX-DRMs we prepend a step of DRM extraction followed by lysis of the pellet for 2 h in PBS containing 1% of Triton X-100 supplemented with a mixture of proteinase inhibitors. Afterwards the pellet and supernatant containing Lubrol WX-DRMs and soluble proteins respectively were loaded on top of continuous sucrose density gradients.

### Biotinylation of Surface Proteins

One µg/ml Biotin-NHS in PBS was added to PBS-washed LNCaP cells and left on ice for 30 minutes. The cells were then washed with ice-cold PBS, quenched twice with 0.1% bovine serum albumin in PBS for 10 minutes and washed again twice with PBS. Afterwards, internalization of PSMA was induced by activation via antibody cross-linking. Remaining biotin on surface proteins was then cleaved by glutathione (1.5 mg/ml). Cells were washed again twice with PBS, solubilized and DRMs were extracted as described above. Immunoprecipitation with anti-PSMA antibody (D2B) was performed and immunoprecipitates were subjected to SDS/PAGE and western blot analysis. The chemiluminescent detection of biotinylated PSMA was carried out using streptavidin horseradish peroxidase (Amersham).

### 2-dimensional Gel Electrophoresis

After activation of PSMA and DRM extraction protein concentrations of pellet fractions were determined using Bradford protein assay. Then equal amounts of protein (400 µg) were precipitated with ethanol over night at -20°C. After centrifugation (10.000×g, 20 min, 4°C) pellets were air-dried and resuspended in rehydration buffer containing 7 M urea, 4% (v/v) chaps, 2 M thiourea and 18 mM DTT for 2 hours at room temperature followed by isoelectric focussing on ReadyStrip IPG Strips (Bio-Rad). Afterwards strips were used for SDS/PAGE as second dimension. Gels were stained with colloidal Coomassie G-250 and quantified by using ImageJ.

### Statistical Analysis

Statistical analysis of results was performed using GraphPad Prism5 applying the parametric paired or unpaired t test (depending on the experimental setup). Significance was accepted when p<0.05.

## Results

### Dimers of PSMA are Associated with Lubrol WX-DRMs

**Figure 1:**
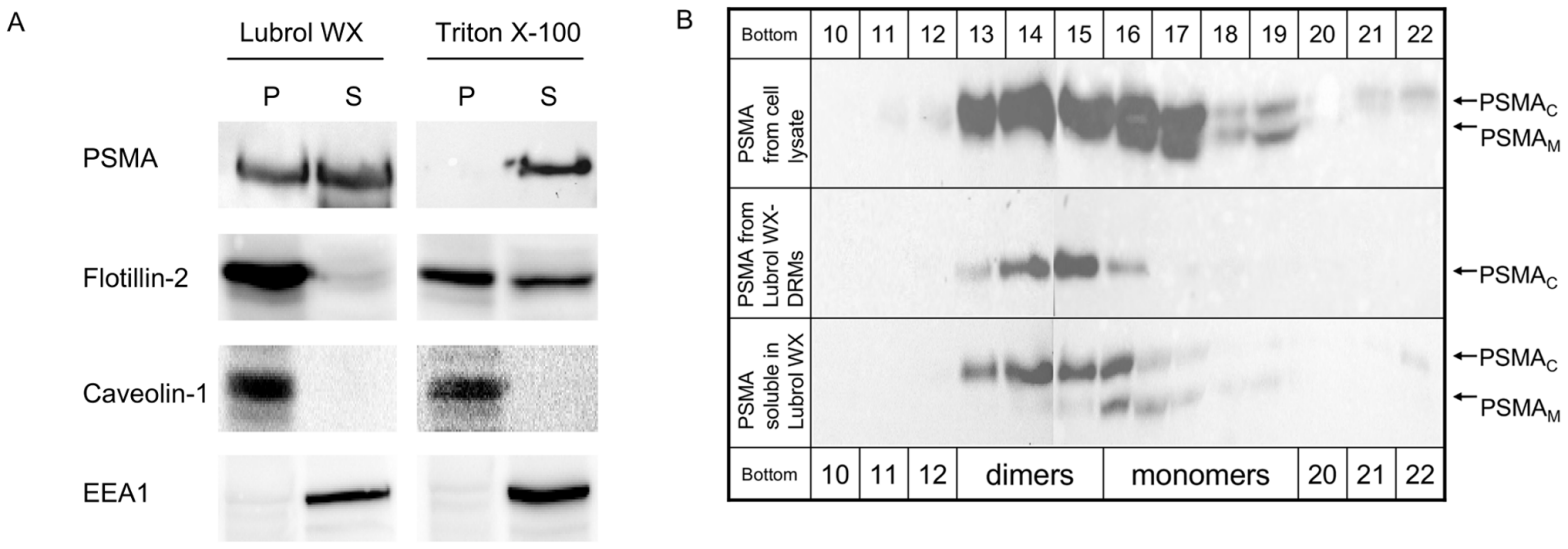
PSMA associates with Lubrol WX-DRMs as a dimeric protein. (A) LNCaP cells were solubilized with either 1% Lubrol WX or 1% Triton X-100 (w/v) in PBS. Lysates were subjected to centrifugation at 100.000×g for 90 min in order to separate the insoluble (P) DRM-fraction and the soluble (S) material. After centrifugation the insoluble fraction (P) was solubilized with a lysis-buffer containing 0.5% (w/v) Triton X-100 and 0.5% (w/v) sodium-deoxycholate and equal amounts of total protein from both fractions were loaded for each lane on SDS gels and blotted. The figure shows that PSMA is completely soluble in Triton X-100 and is partially insoluble in Lubrol WX. To verify these DRMs flotillin-2 and caveolin-1 were used as raft marker proteins and the early endosomal antigen 1 (EEA1) was utilized as a non-raft marker. P pellet, S supernatant (B) After centrifugation the insoluble fraction (P) was solubilized with 1% (w/v) Triton X-100 and 1 ml of both insoluble (P) and soluble (S) fraction was loaded on top of a 5–25% continuous sucrose gradient. After 18 h of centrifugation at 100.000×g, 24 fractions of 500 µl each were collected from bottom to top. Half of each fraction was precipitated using ethanol and PSMA was detected by western blot analysis. The figure shows monomeric PSMA peaking in fractions 16–19 where the mannose-rich glycosylated form (PSMA_M_) can be distinguished from its complex glycosylated counterpart (PSMA_C_). Dimers of PSMA are located in fractions 13–15 that exclusively contain complex glycosylated PSMA (PSMA_C_). PSMA in Lubrol WX-DRMs migrates like the dimeric form of PSMA whereas detergent-soluble PSMA is distributed in later fractions (16 and 17) that contain monomeric PSMA.

LNCaP cells were solubilized with two different detergents, Lubrol WX and Triton X-100, and DRMs were extracted. To verify the isolated DRMs we analyzed the distribution of different lipid raft and non-raft markers within the soluble and non-soluble fractions (Fig. 1A). As raft markers we used flotillin-2 and caveolin-1 which were detected in the pellet fractions of both Lubrol WX- and Triton X-100-DRMs. Flotillin was also partially found in the soluble fractions of Triton X-100-DRMs. As a non-raft marker we utilized the early endosomal antigen 1 (EEA1), which was completely solubilized by Lubrol WX as well as Triton X-100. These results clearly confirm the characteristics of the two types of preparations in being DRMs or non-DRMs. As previously reported [21] mature PSMA is completely soluble in Triton X-100 and is partially insoluble in Lubrol WX (Fig. 1A). Castelletti et al. [21] could also show that as soon as PSMA enters the Golgi, it associates with Lubrol WX-DRMs and this interaction is either maintained or renewed once it reaches the plasma membrane. Homodimerization is also taking place in the Golgi [21], but so far nothing is known about the quaternary structure of PSMA associated with these special types of Lubrol WX-DRMs.

To investigate this aspect, we performed continuous sucrose gradients (5–25%) based on the ability of dimers to migrate to fractions with higher sucrose density than monomers.

As shown in Fig. 1B (also previously shown in ref. [21]) the monomeric form of PSMA peaks in fractions 16–19 where the mannose-rich glycosylated form (PSMA_M_) can be distinguished from its complex glycosylated counterpart (PSMA_C_). Dimers of PSMA are found in fractions 13–15 that exclusively contain complex glycosylated PSMA (PSMA_C_).

To analyze the quaternary structure of PSMA in Lubrol WX-DRMs, cells were first solubilized in Lubrol-WX and DRMs were extracted by ultracentrifugation. After extraction of the insoluble Lubrol WX-DRMs (P) and the soluble (S) material, these were individually loaded on top of a 5–25% continuous sucrose gradient.

PSMA in Lubrol WX-DRMs migrates like the dimeric form of PSMA, peaking in fractions 14 and 15. Detergent-soluble PSMA was distributed in the later fractions (16 and 17), which contain both the mannose-rich and the complex glycosylated forms in their monomeric state. Taken together, it can be concluded that homodimerization of PSMA is required for its association with Lubrol WX-DRMs.

### Appearance of PSMA in Triton X-100-DRMs upon Activation

Upon ligand or antibody cross-linking, some plasma membrane receptors undergo enhanced partitioning into sphingolipid-cholesterol membrane microdomains as an obligatory first step toward participation in early signal transduction events [23], [24], [25]. Experimentally, these microdomains can be isolated based on their insolubility in cold, non ionic detergents [26]. Upon solubilization of cellular extracts with a detergent, detergent-resistant membranes (DRMs) can be recovered by ultracentrifugation or in the floating fractions of sucrose-gradients. For PSMA both procedures revealed comparable results, so that we utilized here the ultracentrifugation approach through out.

**Figure 2:**
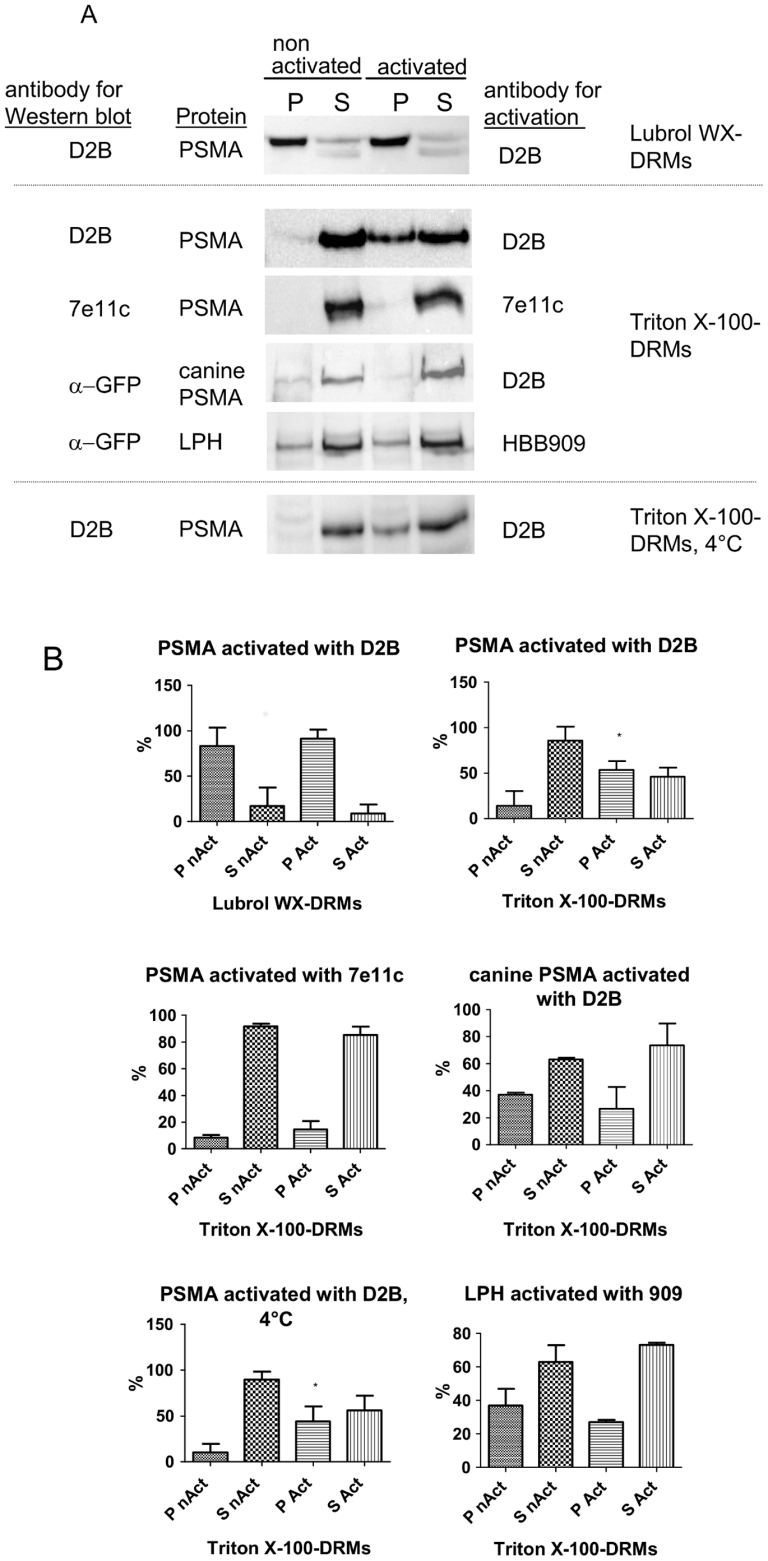
PSMA associates with Triton X-100-DRMs upon activation. (A) LNCaP cells were activated by antibody-induced cross-linking of PSMA at 37°C or 4°C. Subsequently non activated and activated cells were solubilized with either 1% Lubrol WX or 1% Triton X-100 (w/v) in PBS. DRMs (P) and soluble (S) fractions were isolated as described in Fig. 1A. Equal amounts of total protein from all fractions were loaded for each lane on SDS gels and blotted. The distribution of PSMA activated with 7e11c mAb, canine PSMA activated with D2B mAb as well as LPH activated with 909 mAb were analyzed as negative controls. Western blots were developed using D2B mAb for PSMA and anti-GFP mAb for canine PSMA and LPH. The activation of PSMA by antibody-induced cross-linking results in redistribution of PSMA to Triton X-100-DRMs whereas the negative controls show no changes in their detergent solubility properties. P pellet, S supernatant (B) Statistical analysis of results from five independent experiments was performed using GraphPad Prism5 applying the parametric paired t test (two-tailed). Significance was accepted when p<0.05. P nAct - pellet of non activated cells, S nAct - supernatant of non activated cells, P Act - pellet of activated cells, S Act - supernatant of activated cells.

**Figure 3:**
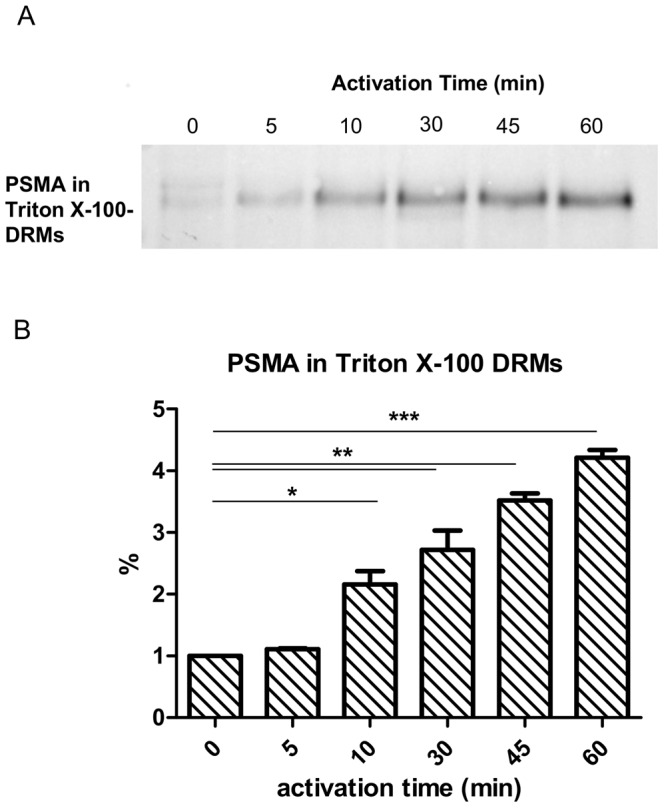
Time course of association of PSMA with Triton X-100-DRMs. (A) LNCaP cells were activated by antibody-induced cross-linking of PSMA for different time periods. Subsequently non activated and activated cells were solubilized with 1% (w/v) Triton X-100 in PBS. Lysates were subjected to centrifugation at 100.000×g for 90 min in order to separate the insoluble (P) DRM-fractions. Equal amounts of total protein from each fraction were further analyzed by western blotting. The figure shows that redistribution of PSMA to Triton X-100-DRMs occurs in a time-dependent manner. (B) Statistical analysis of results from three independent experiments was performed using GraphPad Prism5 applying the parametric paired t test. Significance was accepted when p<0.05.

LNCaP cells were activated by antibody-induced cross-linking and then Lubrol WX- as well as Triton X-100-DRMs were extracted. The process of antibody-induced cross-linking with mAb D2B is henceforth denominated “activation” of PSMA. Indeed, cross-linking of PSMA with antibodies leads to activation of intracellular pathways [22]. As previously reported [21] non activated mature PSMA is mainly insoluble in Lubrol WX and does not associate with Triton X-100-DRMs (Fig. 1A). Strikingly, activation of PSMA by antibody-induced cross-linking results in redistribution of PSMA to Triton X-100-DRMs (Fig. 2A and 2B). To underline the specificity of PSMA clustering in DRMs upon activation we followed three approaches. In the first we used LNCaP cells in conjunction with an antibody that is directed against the cytoplasmic tail of PSMA (7e11c), i.e. do not bind the extracellular luminal part of the protein. The second approach employed mAb D2B in MDCK cells stably expressing canine PSMA. This antibody does not recognize the canine PSMA. Finally we used CHO cells that stably express intestinal lactase phlorizin hydrolase (LPH) that does not undergo internalization and utilized a mAb anti-LPH antibody that is directed against an epitope on the luminal domain of this protein. In all these control experiments neither clustering nor association of PSMA, canine PSMA and LPH with Triton X-100-DRMs could be detected (Fig. 2A and 2B). The clustering and subsequent association of PSMA with Triton X-100-DRMs occurs in a time-dependent manner as shown in Fig. 3. In fact, increased levels of PSMA are recovered in DRMs with increasing time points and reach a plateau between 45 and 60 min.

To examine a possible correlation between clustering into Triton X-100-DRMs and internalization of PSMA after antibody cross-linking we performed the activation assay at 4°C where internalization is blocked. Redistribution of PSMA to Triton X-100-DRMs occurs also after activation at 4°C (Fig. 2A, lower panel). Therefore distribution of PSMA into Triton X-100-DRMs is likely to be independent of internalization.

In conclusion, antibody-induced cross-linking of PSMA at the surface results in a major change in its detergent solubility properties and addressed in what follows is the role of DRMs during activation of PSMA.

### Internalization of PSMA is Mediated by its Association with DRMs

**Figure 4:**
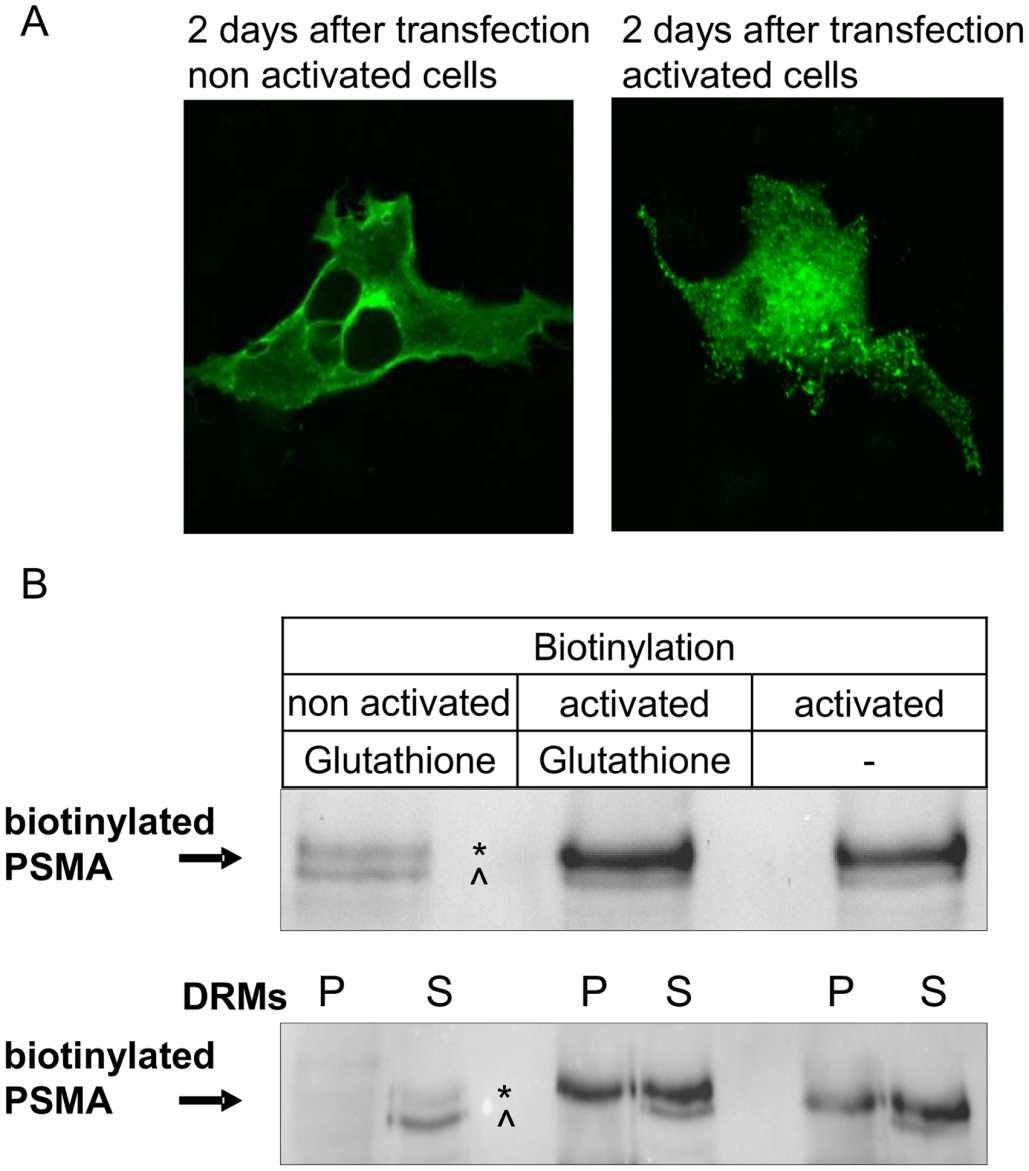
Internalization of PSMA is followed by its association with Triton X-100-DRMs. (A) COS-1 cells were transiently transfected with cDNA encoding YFP-PSMA. Two days after transfection cells were either activated by antibody-induced cross-linking of PSMA or directly visualized using a Leica TCS SP5 confocal laser microscope. The figure shows that PSMA is internalized and appears in vesicular structures after activation with antibody cross-linking. (B) LNCaP cells were cell-surface biotinylated for 30 min on ice. Cells were washed and quenched twice with 0.1% bovine serum albumin. Afterwards, internalization of PSMA was induced by antibody cross-linking. Remaining biotin on surface proteins was then cleaved by glutathione. Cells were washed again, solubilized with Triton X-100 and DRMs were extracted. After centrifugation the insoluble fraction (P) was solubilized with a lysis-buffer containing 0.5% (w/v) Triton X-100 and 0.5% (w/v) sodium-deoxycholate and PSMA was immunoprecipitated from both insoluble (P) and soluble (S) fractions. Immunoprecipitates were subjected to SDS/PAGE and western blot analysis. The chemiluminescent detection of biotinylated PSMA was carried out using streptavidin horseradish peroxidase. The blots confirm substantial internalization of PSMA upon antibody-induced cross-linking and the internalized PSMA in the antibody-activated samples was localized in Triton X-100-DRMs in contrast to PSMA in the non-activated cells. P pellet, S supernatant * PSMA_C_ ∧ PSMA_M._

The clustering of PSMA occurs already at the cell surface. We wanted to determine whether this event is required for internalization. Fig. 4A shows that PSMA is internalized and appears in vesicular structures after activation with antibody cross-linking at the cell surface. To confirm these data at the protein level the plasma membrane of LNCaP cells was biotinylated and internalization of PSMA was induced by antibody cross-linking. Fig. 4B (upper panel) demonstrates a strong protein band corresponding to internalized PSMA in activated cells. The glutathione-treated and non-treated activated cells revealed almost similar band intensity of mature PSMA indicating that the internalization of PSMA is almost complete. On the other hand the non activated cells revealed faint bands corresponding to complex (PSMA_C_) as well as mannose-rich glycosylated PSMA (PSMA_M_). This is likely due to (i) slight diffusion of biotin through the membrane thus labelling both intracellular forms of PSMA or (ii) to incomplete action of glutathione. Nevertheless this does not alter the significance of this result, which clearly demonstrates that PSMA undergoes substantial internalization upon antibody-induced activation.

To investigate the role of Triton X-100-DRMs during internalization DRM extraction was performed. The data (Fig. 4B, lower panel) clearly show that biotinylated PSMA in the antibody-activated samples was localized in Triton X-100-DRMs in contrast to PSMA in the non-activated cells. Therefore activation of PSMA results in a drastic shift in the detergent solubility properties of both surface PSMA and internalized PSMA. Again, the supernatant of non activated cells shows a faint band of lower molecular weight likely corresponding to the high-mannose glycosylated form of PSMA (PSMA_M_) which is presumably due to slight diffusion of biotin through the membrane.

### Identification of Candidate Proteins Potentially Interacting with PSMA in DRMs

**Figure 5:**
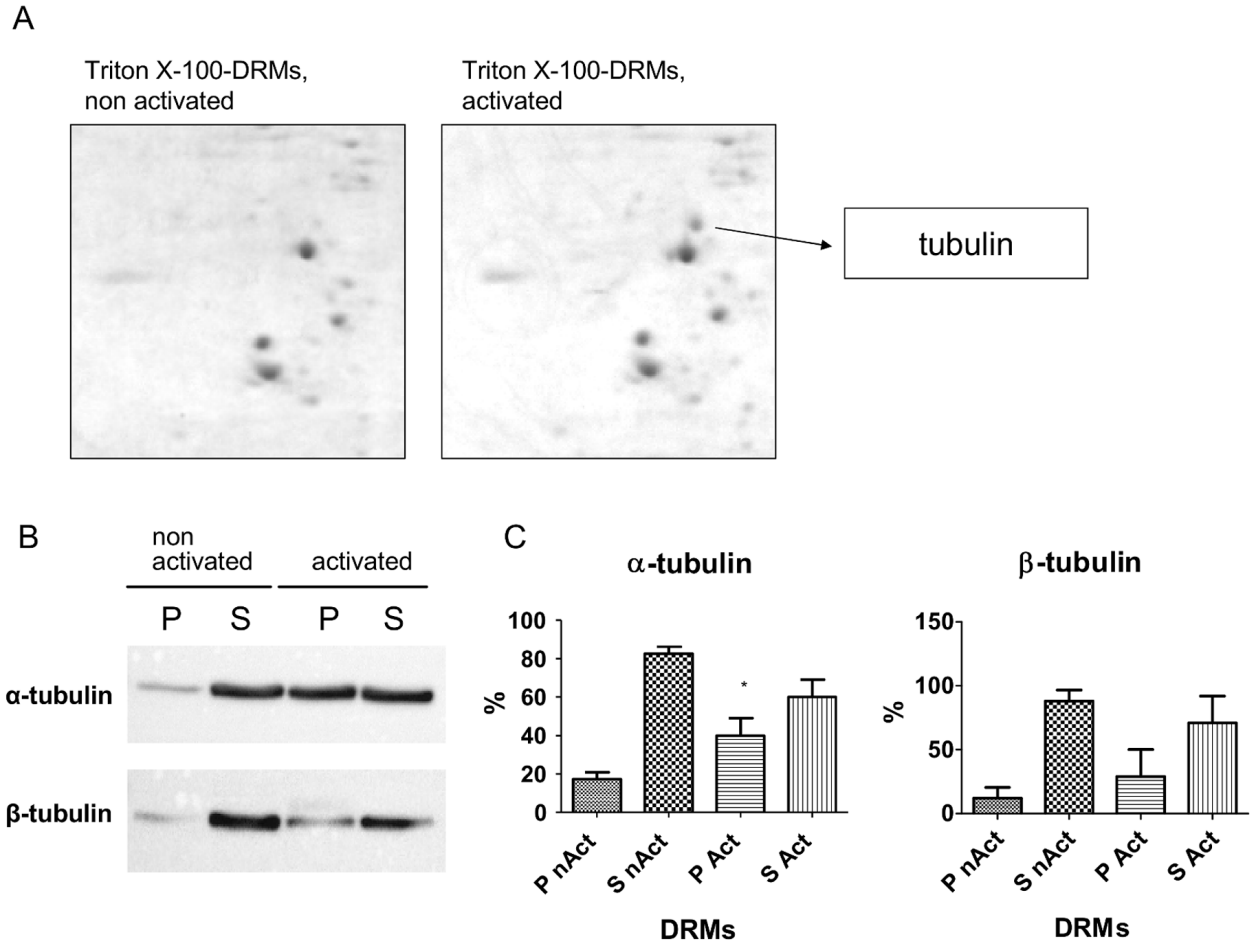
Identification of proteins potentially interacting with PSMA. (A) LNCaP cells were either activated by antibody-induced cross-linking of PSMA or subsequently solubilized with 1% Triton X-100 and DRMs were extracted. After centrifugation the insoluble fraction (P) was solubilized with a lysis-buffer containing 0.5% (w/v) Triton X-100 and 0.5% (w/v) sodium-deoxycholate and equal amounts of protein (400 µg) were precipitated with ethanol over night. Pellets were resuspended in rehydration buffer and 2-dimensional gel electrophoresis was performed. Gels were stained with colloidal coomassie G-250 and protein spots were quantified by using ImageJ. The 2-D gels show variations in the expression levels of different proteins in Triton X-100-DRMs due to antibody-induced activation of PSMA. One of these proteins is tubulin that increased substantially in Triton X-100-DRMs isolated from activated cells. (B) LNCaP cells were either activated by antibody-induced cross-linking of PSMA or subsequently solubilized with 1% Triton X-100 and DRMs were extracted. After centrifugation the insoluble fraction (P) was solubilized with a lysis-buffer containing 0.5% (w/v) Triton X-100 and 0.5% (w/v) sodium-deoxycholate. Equal amounts of total protein from all fractions were loaded for each lane on SDS gels and α- as well as β-tubulin concentrations were detected by western blot analysis. The blots confirm the redistribution of α-tubulin as well as β-tubulin to Triton X-100-DRMs upon activation of PSMA. P pellet, S supernatant (C) Statistical analysis of results from three independent experiments was performed using GraphPad Prism5 applying the parametric paired t test (two-tailed). Significance was considered at p<0.05. P nAct - pellet of non activated cells, S nAct - supernatant of non activated cells, P Act - pellet of activated cells, S Act - supernatant of activated cells.

Antibody-induced internalization of PSMA is involved in signalling processes [22] and interactions of PSMA with different proteins like the actin cross-linking protein filamin A [16] and clathrin as well as adaptor protein complex-2 [27] have been shown to participate in internalization of PSMA. To identify additional proteins potentially interacting with PSMA the Triton X-100-DRMs of non activated cells were compared with those of activated cells by 2D-gel electrophoresis. Mean relative spot volumes and differences in expression were calculated using ImageJ and the differing spots were analyzed by mass spectrometry followed by Swiss-Prot database search with MASCOT to assign identities. As shown in Fig. 5A the expression levels of a number of proteins varied in Triton X-100-DRMs due to antibody-induced activation of PSMA. One of these proteins is tubulin that increased substantially in Triton X-100-DRMs isolated from activated cells.

To confirm this finding by western blot analysis LNCaP cells were activated or non-activated by antibody-induced cross-linking followed by Triton X-100-DRM extraction. Fig. 5B and 5C shows a significant clustering of α-tubulin in Triton X-100-DRMs upon activation. Also the levels of β-tubulin increase in these DRMs, yet not to the same extent as α-tubulin (Fig. 5B and C). These findings strongly suggest that microtubules are involved in internalization of PSMA.

**Figure 6:**
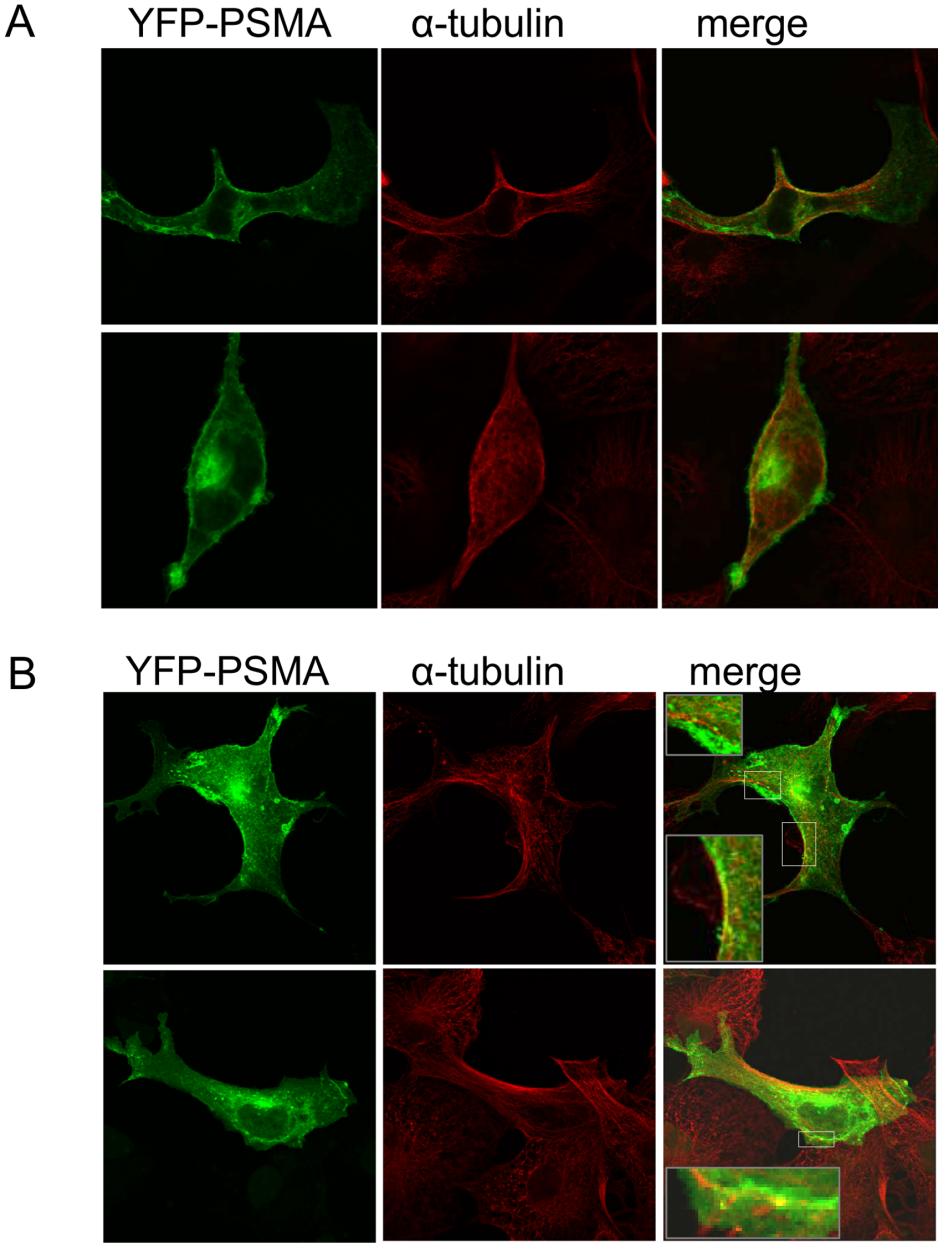
Internalization of PSMA along microtubules. (A) COS-1 cells were transiently transfected with cDNA encoding YFP-PSMA. Two days after transfection cells were fixed and α-tubulin was detected by indirect immunofluorescence. In these cells α-tubulin did not colocalize with YFP-PSMA. (B) COS-1 cells were transiently transfected with cDNA encoding YFP-PSMA. Two days after transfection cells were activated by antibody-induced cross-linking of PSMA, fixed and α-tubulin was detected by indirect immunofluorescence. The figure shows internalization of PSMA upon activation and several endosomes containing YFP-PSMA colocalize with α-tubulin.

The colocalization of PSMA with α-tubulin before and after cross-linking and internalization was examined in COS-1 cells that transiently express the YFP-tagged form of PSMA. Fig. 6A revealed YFP-PSMA in the Golgi apparatus and at the cell surface in non-activated COS-1 cells. α-tubulin that was detected by indirect immunofluorescence did not colocalize with YFP-PSMA in these cells. Upon antibody-induced cross-linking of PSMA internalization occurred and several endosomes containing YFP-PSMA can be observed to colocalize with α-tubulin along or in close vicinity to the cell surface (Fig. 6B). These observations together suggest that YFP-PSMA is transported along the tubulin filaments during internalization.

## Discussion

**Figure 7:**
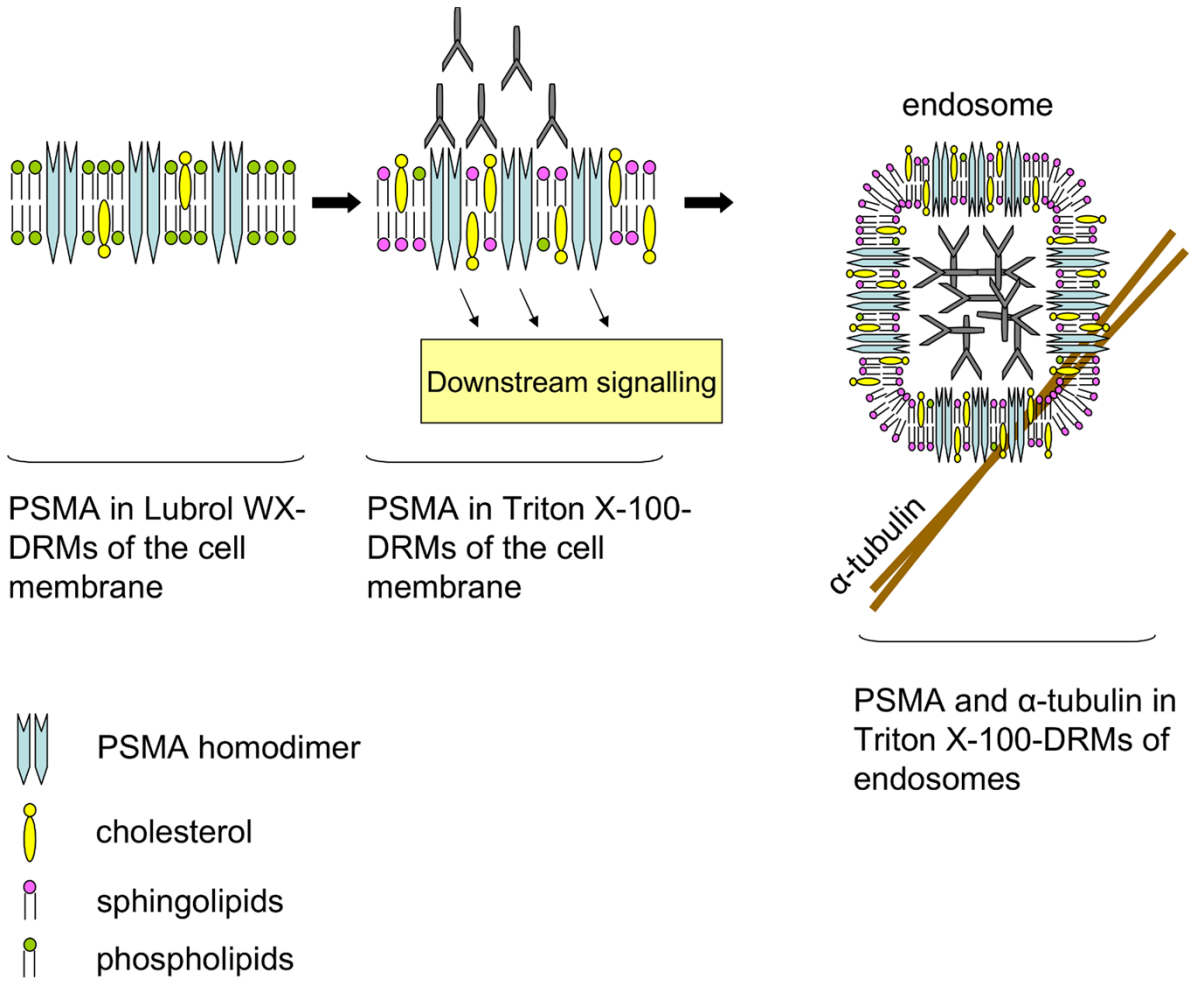
Schematic presentation of DRM-associated internalization of PSMA. PSMA is transported to the Golgi where it associates with Lubrol WX-DRMs. Likewise, homodimeric forms as well as cell surface forms of PSMA associate with similar type of DRMs at the cell surface. Antibody-induced cross-linking of PSMA homodimers results in a partitioning of PSMA and α-tubulin into Triton X-100-DRMs. Concomitantly internalization of PSMA occurs along α-tubulin filaments.

PSMA is a potential target for immunotherapy of prostate cancer [15], [28], as well as for other histotypes of solid tumors due to its de novo expression in tumor neovasculature [8], [9]. In this manuscript we addressed the role of lipid rafts in two major physiological events that are of pivotal importance in the life cycle of PSMA, homodimerization and internalization. Homodimerization of PSMA is required for its enzymatic activity [29] and occurs late along the secretory pathway [21]. Our results demonstrate that the transition from monomeric to dimeric state is linked to the formation of a complex glycosylated form of PSMA and to the association of the formed PSMA homodimers with Lubrol WX-DRMs. Other biosynthetic forms of PSMA, such as the mannose-rich monomeric form favour a different membrane environment that is reflected by their association with Tween 20-DRMs in the ER and prior to their exit from this organelle [21]. The dimeric form of PSMA is retained in Lubrol WX-DRMs at the cell surface [21] suggesting that dimerization and association with this type of DRMs are important for a full biological activity of PSMA particularly towards its interaction with its natural ligand. This interaction is mimicked in our study by cross-linking of homodimeric PSMA at the cell surface with anti-PSMA antibodies. As a consequence two major dramatic events occur: The first is an efficient internalization of PSMA with an almost complete disappearance of PSMA from the cell surface and appearance as discrete intracellular spots; the second is a shift of dimeric PSMA from Lubrol WX-DRMs to a different membrane environment that is enriched in cholesterol and sphingolipids and exemplified by Triton X-100-DRMs or lipid rafts. A major component that is associated with these rafts and PSMA is α-tubulin. In endothelial cells internalization of PSMA has been shown to implicate its interaction with caveolin 1 [17] thus suggesting that lipid rafts may play a role in this event since caveolin is a major structural protein of caveolae and these in turn represent a special type of lipid rafts. In line with these observations our time-dependent endocytosis of PSMA provides an unequivocal support to the notion that lipid rafts are directly implicated in the clustering and internalization of PSMA (Fig. 7).

While cross-linking of PSMA with antibodies and eliciting internalization could be considered to be an artificial system, cross-linking of membrane proteins is normally a physiological phenomenon that can lead to redistribution of these proteins into lipid rafts, resulting in novel protein interactions and initiation of cell signalling [24], [30]. Although occurring naturally via multivalent ligands, similar responses have been observed using antibodies [24]. The natural ligands of PSMA in prostate cancer are still not known. Under normal conditions PSMA hydrolyzes its two recognized natural substrates, N-acetyl- aspartyl-glutamate (NAAG) and folyl-poly-γ-glutamates. This could be shown for the nervous system and small intestine, where PSMA directly participates in signal transmission via neural pathways and intestinal folate absorption, respectively [31]. Interestingly PSMA is highly homologous to NAALADase (*N*-acetylated α-linked l-amino dipeptidase) which is specifically characterized by its ability to hydrolyze the neuropeptide NAAG [32]. In contrast to NAALADase which has been extensively studied due to its presumed regulatory role in glutamate neurotransmission, the role of PSMA and its natural ligands in prostate cancer still remain unknown.

In previous studies we could demonstrate that the small GTPases RAS and RAC1 and the MAPKs p38 and ERK1/2 are activated during this process of antibody-induced activation. As downstream effects we observed a strong induction of NF-kB activation associated with an increased expression of IL-6 and CCL5 genes and that IL-6 and CCL5 enhanced the proliferative potential of LNCaP cells synergistically [22]. While PSMA favours a special type of lipid rafts and thereby triggers IL-6 and CCL5 expression, PSMA mobilization could be one of the pletora of inside-out signals continuously controlling the basal expression of cytokines and chemokines, whereas the strong remodelling of the plasma membrane induced by ligand encounters or antibody treatment may act as waves of outside-in signals.

Along these lines we strongly propose an essential role for lipid rafts or DRMs in endocytosis and signalling pathways involving PSMA.

Comparable effects are already described for the Myelin Oligodendrocyte Glycoprotein [33], [34] and the T-cell receptor [33]. In both cases antibody-induced cross-linking leads to redistribution to Triton X-100-DRMs coupled to downstream signalling cascades.

The microtubule cytoskeleton is particularly important for polarized targeting of apical cargo. Microtubule depolymerization results in aberrant delivery of several apical proteins, including PSMA, to the basolateral surface [35]. But until present few information, if any, is available on the role of microtubules during internalization of PSMA. We could show that α-tubulin as well as partially β-tubulin are together with PSMA redistributed to Triton X-100-DRMs upon activation of PSMA by antibody-induced cross-linking. Immunofluorescence revealed that PSMA undergoes internalization along α-tubulin, suggesting the necessity of microtubules for internalization of PSMA. The internalization of PSMA involves its interaction with filamin A, an actin cross linking protein, and this association is involved in the localization of PSMA to the recycling endosomal compartment [16]. Our data extends the role played by the cytoskeleton to include α-tubulin clearly indicating that microtubules are key players in PSMA endocytosis (Fig. 7).

In essence, understanding the molecular mechanisms underlying the antibody-induced cross-linking, deciphering the membrane structures and components governing this event as well as the internalization of PSMA constitute essential prerequisites to understand its role in the most aggressive and metastasizing forms of cancer and for its utilization as a therapeutically suitable target in prostate cancer.
